# GeNIS: A modular dataset for network intrusion detection and classification

**DOI:** 10.1016/j.dib.2025.111487

**Published:** 2025-03-21

**Authors:** Miguel Silva, Daniela Pinto, João Vitorino, José Gonçalves, Eva Maia, Isabel Praça

**Affiliations:** Research Group on Intelligent Engineering and Computing for Advanced Innovation and Development (GECAD), School of Engineering, Polytechnic of Porto (ISEP/IPP), 4249-015 Porto, Portugal

**Keywords:** Network flow, Packet capture, Attack classification, Anomaly detection, Machine learning, Cybersecurity, Dataset

## Abstract

The development of artificial intelligence solutions for cyberattack detection and classification require high-quality and representative data. However, there is a scarcity of labelled datasets focused on the cyberattacks that target vulnerable small and medium-sized enterprises. To allow organizations to improve their intrusion detection systems according to their types of users, their active services, and the network protocols they use, it is necessary to provide reliable captures of different types of benign and malicious traffic. The GECAD Network Intrusion Scenarios (GeNIS) dataset contains multiple sequential attack scenarios and different types of realistic normal network activity, recorded during advanced network simulations on the Airbus CyberRange platform. The raw network packets were analyzed to generate labelled network flows, with the computation of statistical features to represent the traffic patterns of local and remote attackers, normal users and administrators, and background traffic of an enterprise computer network. GeNIS follows a modular design, providing raw packet capture next generation (PCAPNG) files with over 37 million packets of each intermediate attack step to enable an in-depth analysis with different flow exporters, feature extraction, and feature selection tools, as well as filtered CSV files with over 2.8 million flows created with 5, 10, 30, and 60 s flow intervals. The flows were preprocessed to provide a reliable benchmark dataset with the most relevant features for the training, validation, and testing of robust machine learning and deep learning models.

Specifications Table

**Table:** 

Subject	Computer Sciences
Specific subject area	Network intrusion detection and cyberattack classification with packet captures and network flows for machine learning and deep learning models
Type of data	Packet capture, Table Raw, Filtered, Processed
Data collection	Raw packet capture next generation (PCAPNG) files were collected by performing advanced network simulations on the Airbus CyberRange platform, which was used as a cybersecurity testbed, and recording them using the dumpcap tool. Sequential attack scenarios were executed following a modular design, using custom scripts with open-access libraries, while normal network activity was generated using a custom configuration of the Benign User Profiler (BUP) tool. Preprocessed comma-separated value (CSV) files were created by automatically analyzing the packets with the previously developed Holistic nEtwork featuRes Aggregator (HERA) tool, which was configured to generate labelled network flows, computing standard statistical features to represent the traffic patterns.
Data source location	School of Engineering, Polytechnic of Porto, Portugal
Data accessibility	Repository name: Zenodo Data identification number: 10.5281/zenodo.14919237 Direct URL to data: https://doi.org/10.5281/zenodo.14919237 [1]
Related research article	D. Pinto, I. Amorim, E. Maia, and I. Praça, “A novel approach to network traffic analysis: the HERA tool”, 23rd IEEE Int. Conf. on Trust, Security and Privacy in Computing and Communications (TrustCom), 2024. doi: 10.48550/arXiv.2501.07475.

## 1 Value of the Data

• This dataset provides multiple sequential attack scenarios and realistic normal network activity recorded on the Airbus CyberRange platform, to improve the detection and classification of cyberattacks targeting vulnerable small and medium-sized enterprises.
• The network traffic is provided in a modular design, with separate captures of each intermediate attack step to enable the use of specific subsets for different organizations, according to their types of users, their active services, and the network protocols they use.
• The raw PCAPNG data contains the complete record of all 37.681.001 transmitted network packets organized according to attack step to enable a more in-depth analysis and validation with different flow exporters, feature extraction, and feature selection tools.
• The preprocessed CSV data contains statistical features of 2.806.168 network flows with 5 to 60 s flow intervals that represent the traffic patterns of local and remote attackers, normal users and administrators, and background traffic of an enterprise computer network.
• Researchers, artificial intelligence engineers, and cybersecurity professionals can benefit from this dataset to train, validate, and test their machine learning and deep learning solutions for intelligent network intrusion detection systems.

## 2 Background

The integration of artificial intelligence in network intrusion detection systems is a promising approach to identify suspicious network activity [2]. Nonetheless, for these solutions to be reliable and robust, it is necessary to train them with high-quality and representative data of different types of benign and malicious traffic [3].

Early efforts on the creation of network intrusion detection datasets relied on the collection of raw packet traces from simple experimental topologies, which often had limited scalability and realism, as they lacked important data due to privacy concerns [4]. To overcome these limitations, network simulation platforms, designated as CyberRanges, are starting to be used to perform more sophisticated attack scenarios in a controlled environment, replicating complex network infrastructures of real-world enterprises [5].

However, despite ongoing efforts to improve traffic diversity and extract relevant statistical features, most public datasets only contain standalone attacks and use their own flow exporters, which results in network flows with incompatible features that limit the integration of multiple datasets [6]. As attack vectors continue to become more sophisticated, it is essential to make available modular datasets with separate captures for each sequential step of cyberattack, to enable organizations to use specific subsets with different flow intervals according to their needs.

## 3 Data Description

This dataset follows a modular design, separating the recorded network traffic into multiple folders to provide raw, filtered, and preprocessed versions that can be used independently or combined with each other for a more in-depth analysis.

The recorded traffic was organized into hierarchical labels, from the most general ‘0’ and ‘1’ labels representing benign and malicious traffic, to the more specific categories and subcategories of each specific attack or benign activity profile. Table 1 presents an overview of the considered class labels. The experimental design and methods used to create and collect the traffic of each subcategory will be further detailed in the next section.

**Table 1:** Network flow class labels.

Binary Label	Category Label	SubCategory Label
0	benign	benign-admin
		benign-background
		benign-user
1	bruteforce	bruteforce-ftp
		bruteforce-smb
		bruteforce-ssh
	dos	dos-hulk
		dos-icmp
		dos-pushack
		dos-slowloris
		dos-udp
	recon	recon-dns
		recon-nmap

To ensure a good usability of the dataset, the recorded packets and network traffic flows were divided into five main folders. The following subsections detail the files of each folder:

• *0-info* – The general information regarding the dataset.
• *1-packets* – The raw packets, organized by subcategory label.
• *2-flows* – The labelled flows, organized by subcategory label.
• *3-scenarios* – The labelled flows, filtered by sequential attack scenario.
• *4-preprocessed* – The preprocessed flows ready for use.

### 3.1 Info folder

The *0-info* folder contains a list of all the features used in this dataset, including their feature type and a description. Furthermore, to ensure transparency and reproducibility, it also includes the ground truth used for labelling the network flows. It includes the following files:

• *genis-features.csv* – List of all considered features, with respective descriptions.
• *genis-ground-truth.csv* – List of ground truth labels for each capture, with start and end times.

### 3.2 Packets folder

The *1-packets* folder contains all the raw packets in standard PCAPNG files to ensure that they can be opened with publicly available network packet analyser tools. It contains the following files:

• *attack-bruteforce-ssh-ftp.pcapng* – Packets of SSH and FTP Bruteforce attacks.
• *attack-bruteforce-smb.pcapng* – Packets of SMB Bruteforce attacks.
• *attack-bruteforce-ssh.pcapng* – Packets of SSH Bruteforce attacks.
• *attack-dos-hulk.pcapng* – Packets of Hulk attacks.
• *attack-dos-icmp.pcapng* – Packets of ICMP Flood attacks.
• *attack-dos-pushack.pcapng* – Packets of Push&Ack attacks.
• *attack-dos-slowloris.pcapng* – Packets of Slowloris attacks.
• *attack-dos-udp.pcapng* – Packets of UDP Flood attacks.
• *benign-admin-activity.pcapng* – Packets of Administrator activity.
• *benign-background-activity.pcapng* – Packets of Background activity.
• *benign-user-activity.pcapng* – Packets of User activity.

### 3.3 Flows folder

The *2-flows* folder contains all the labelled network flows in a tabular data format in standard CSV files that can be easily compressed, imported, and exported. It is divided into 4 subfolders:

• *flows-5-sec* – All 5-s labelled flows.
• *flows-10-sec* – All 10-s labelled flows.
• *flows-30-sec* – All 30-s labelled flows.
• *flows-60-sec* – All 60-s labelled flows.

Each of the subfolders contains flows that were generated from the same raw PCAPNG files, but with a different flow interval, which will be further detailed in the next section. Therefore, they contain different versions of the following files:

• *attack-bruteforce-ftp.csv* – Flows of FTP Bruteforce attacks.
• *attack-bruteforce-smb.csv* – Flows of SMB Bruteforce attacks.
• *attack-bruteforce-ssh.csv* – Flows of SSH Bruteforce attacks.
• *attack-dos-hulk.csv* – Flows of Hulk attacks.
• *attack-dos-icmp.csv* – Flows of ICMP Flood attacks.
• *attack-dos-pushack.csv* – Flows of Push&Ack attacks.
• *attack-dos-slowloris.csv* – Flows of Slowloris attacks.
• *attack-dos-udp.csv* – Flows of UDP Flood attacks.
• *benign-admin-activity.csv* – Flows of Administrator activity.
• *benign-background-activity.csv* – Flows of Background activity.
• *benign-user-activity.csv* – Flows of User activity.

### 3.4 Scenarios folder

The *3-scenarios* folder contains the network flows filtered according to the sequential attack scenarios and combined with the benign flows of admin, background, and user activity at the appropriates hours of the day. It is also divided into 4 subfolders of CSV files, per flow interval:

• *scenarios-5-sec* – The scenarios with 5-s labelled flows.
• *scenarios-10-sec* – The scenarios with 10-s labelled flows.
• *scenarios-30-sec* – The scenarios with 30-s labelled flows.
• *scenarios-60-sec* – The scenarios with 60-s labelled flows.

Each of the subfolders contains flows that were generated from the same raw packets, but with a different flow interval, which will be further detailed in the next section. Therefore, they contain different versions of the following files:

• *scenario-1-disrupt.csv* – Sequential flows of Scenario 1.
• *scenario-2-disrupt.csv* – Sequential flows of Scenario 2.
• *scenario-3-disrupt.csv* – Sequential flows of Scenario 3.
• *scenario-4-disrupt.csv* – Sequential flows of Scenario 4.
• *scenario-5-disrupt.csv* – Sequential flows of Scenario 5.
• *scenario-6-authtest.csv* – Sequential flows of Scenario 6.
• *scenario-7-authtest.csv* – Sequential flows of Scenario 7.
• *scenario-8-authtest.csv* – Sequential flows of Scenario 8.

### 3.5 Preprocessed folder

The *4-preprocessed* folder contains the preprocessed network flows of all scenarios combined into a training set and a holdout set for each flow interval, created with shuffling and stratification to ensure the correct proportion of all classes. It contains the following CSV files, with a subset of selected features representing the most relevant traffic characteristics:

• *genis-5-sec-train.csv* – Training set of 5-s flows.
• *genis-5-sec-test.csv* – Holdout set of 5-s flows.
• *genis-10-sec-train.csv* – Training set of 10-s flows.
• *genis-10-sec-test.csv* – Holdout set of 10-s flows.
• *genis-30-sec-train.csv* – Training set of 30-s flows.
• *genis-30-sec-test.csv* – Holdout set of 30-s flows.
• *genis-60-sec-train.csv* – Training set of 60-s flows.
• *genis-60-sec-test.csv* – Holdout set of 60-s flows.

## 4 Experimental Design, Materials and Methods

This dataset was developed using the cloud-based network simulation infrastructure that GECAD has available on the Airbus CyberRange platform [7], with a total of 32 GHz of CPU power, 112 GB of RAM and 4 TB of storage. This platform supports the creation of digital twins of real-world computer networks, with the configuration of complex network topologies and the deployment of multiple types of servers and workstations with independent computing and networking capabilities.

To safely perform cyberattacks in a controlled environment, an independent work zone was created to be used as a cybersecurity testbed, replicating a network infrastructure that could be used by small and medium-sized enterprises. The environment included a switched port analyzer (SPAN) that performed port mirroring on multiple network interfaces throughout the network, to send all the relevant traffic to an isolated monitoring machine. The raw traffic was collected using dumpcap [8], a standard tool to capture live traffic and store it sequentially.

The network simulations took place from February 6th to February 12th, 2025. First, benign network activity was recorded on the 6th and 7th, during regular Thursday and Friday workdays, and then common background traffic was captured on the 8th, during the weekend. The cyberattacks were performed and recorded at the start of the following week, from February 10th to 12th. The utilized network infrastructure, the considered scenarios, and the performed feature extraction are detailed in the following subsections.

### 4.1 Infrastructure

In this environment, the captured network traffic represents the activity of a typical business day within an organization. The setup consists of six different Local Area Networks (LANs) designed to reflect typical topologies found in mid-sized organizations. Real-world attack scenarios were conducted, while the generated benign traffic includes a range of activities, from simple interactions to complex operations involving multiple services. This ensures that the dataset is relevant to the analysis of real-world network behaviour.

One of the LANs is the Admin LAN, which is dedicated to system administration and maintenance tasks. It operates on the subnet 192.168.131.0/24 and includes computers and tools used by system administrators to manage and maintain the organization's servers and IT infrastructure.

This network is accessed solely by technical personnel with advanced skills in IT systems and security. These users are active during standard working hours or during maintenance windows, when they perform maintenance tasks on servers. Expected activities in this subnet include system updates, server monitoring, and troubleshooting.

The second LAN, known as the User LAN, is dedicated to general business activities for non-technical employees. It operates on the 192.168.132.0/24 subnet and primarily consists of devices used for tasks such as accessing web applications, email communication, and office productivity. Users on this network frequently connect to external websites and services and do not engage in system administration of the organization's assets. Their activities are restricted to standard business operations during working hours, including document sharing and correspondence.

The Server LAN is reserved for internal servers that support the organization's core IT infrastructure.

It operates on the subnet 192.168.130.0/24 and hosts servers such as the Active Directory (AD), file and email servers which are critical for business operations. Access to this network is limited to system administrators and other authorized personnel. Activity in this subnet typically involves server communications, maintenance, and updates performed by administrators.

The DMZ network is used for systems that must be accessible to external users. It operates on the subnet 192.168.128.0/24 and contains servers hosting public-facing services, such as web servers, email gateways, nameservers, proxy servers and FTP servers. The systems in this network are accessed by both remote employees and external users.

The Remote User Network is dedicated to employees who connect to the corporate infrastructure remotely. It operates on the subnet 192.168.141.0/24 and includes VPN gateways and endpoints used by remote workers to securely access internal resources. This network is accessed exclusively by employees working outside the office, who rely on VPN tunnels to connect securely to the organization's systems. Their activities can include normal business operations, such as accessing files, sending emails, and performing tasks remotely.

**Fig. 1:**
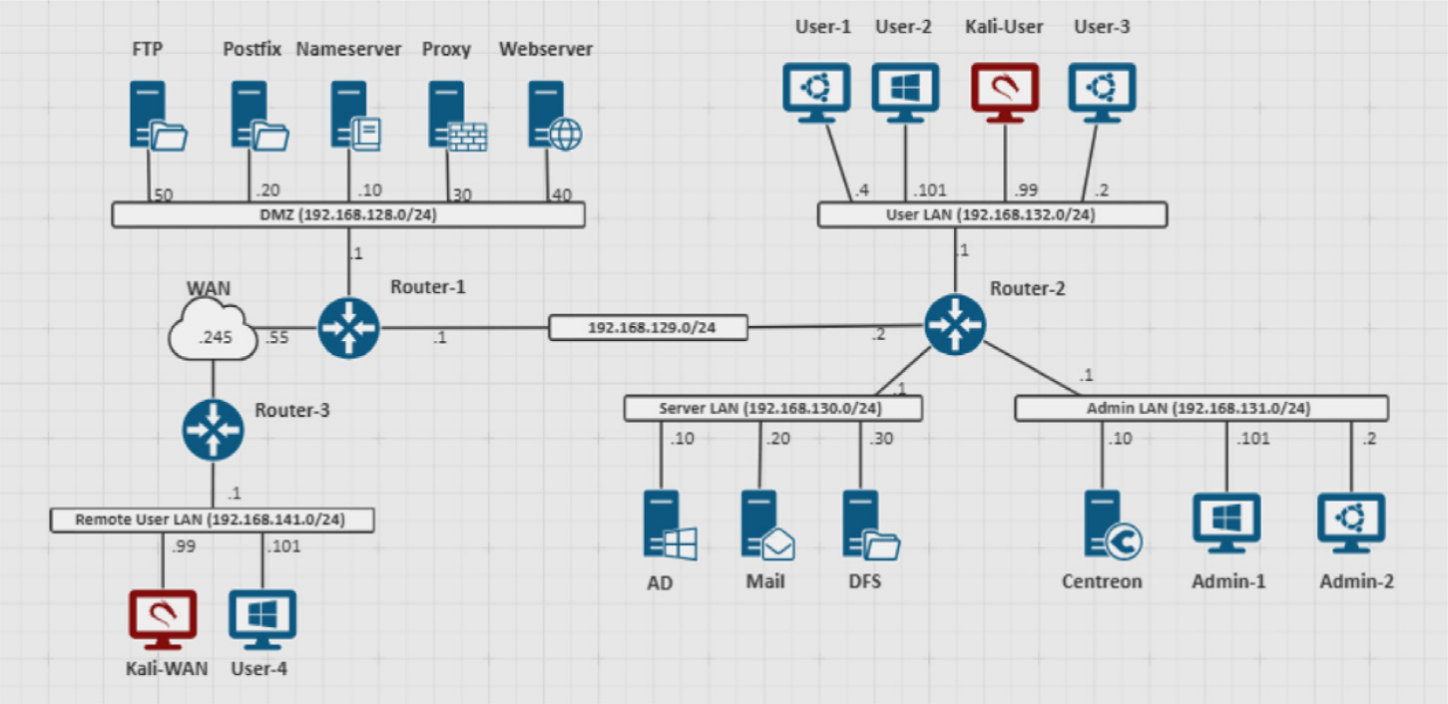
Network topology used for traffic capture.

The network topology described above can be visualized in Fig. 1.

### 4.2 Benign traffic

The benign network traffic was generated using the Benign User Profiler (BUP) [9], a tool published under the GPL-3.0 license. It was specifically designed to generate realistic network activity by scheduling and executing tasks tailored to the characteristics of each subnet. BUP was deployed on three devices within the topology, the User-1 and User-3 devices on the user LAN and the Admin-2 on the admin LAN, to ensure that the activity on each subnet reflected typical user behavior for that network segment. The devices on the two LANs were configured with two different profiles, depending on which LAN the device was on. The profiles configured in BUP include:

• **Profile 1: User Activity (User LAN)**: This profile simulates a typical working day for non-technical employees using the User VLAN. Activities include accessing web applications, sending and receiving emails, and system updates, reflecting the usual behaviors of office workers during their daily tasks.
• **Profile 2: Administrator Activity (Admin LAN)**: This profile simulates a working day for system administrators in the Admin VLAN. Activities include server maintenance activities performed by IT personnel with advanced technical skills.

In profile 1, the two devices configured to use the BUP followed the same traffic generation scenario, offset by a time difference of five minutes. Emails sent from one device were received by the other, creating realistic email communication within the network. The traffic schedule was designed to simulate varying levels of activity, alternating between periods of higher and lower traffic, to better mimic real-world network behavior compared to a simple periodic pattern. For this profile, the time frame considered for a normal employee lunch break was 12:00 to 13:00.

Table 2 presents the traffic schedule for one of the devices that was configured to run profile 1. In addition to the protocols associated with the scheduled activities in this scenario, the dataset also includes machine background traffic, which was passively captured. This ensures a more comprehensive representation of the device's overall network behavior.

**Table 2:** User profile activity schedule.

Hours	Protocol
9:00	IMAP, HTTP
10:00	IMAP, HTTP
11:00	IMAP, HTTP, SMPT
12:00	Update
13:00	IMAP, HTTP, SMTP
14:00	IMAP, HTTP
15:00	IMAP, HTTP, SMTP
16:00	IMAP, HTTP

Profile 2 involves various administrative tasks performed by system administrators. These tasks include uploading files to FTP servers, accessing remote machines via SSH, testing DNS functionality, manually interacting with SMB administrative shares to add or retrieve configuration files, and testing the functionality of web servers. Table 3 provides a summary of the traffic generated by this profile, as configured in the BUP.

**Table 3:** Admin profile activity schedule.

Hours	Protocol
9:00	–
10:00	SSH, FTP, HTTP, SMB
11:00	SSH, DNS
12:00	–
13:00	FTP
14:00	HTTP
15:00	SSH, SMB
16:00	SSH

The traffic generated by the two profiles was captured separately to provide a modular approach for traffic analysis. In addition to the traffic generated by the BUP profiles, there is also background traffic generated by the processes inherent in the operation of the servers and clients. This background traffic includes ARP packets, NBNS packets related to Active Directory functionality, and TCP and DNS traffic. This background traffic was captured separately from the BUP-generated traffic to allow for an analysis of the network traffic prior to applying the BUP.

### 4.3 Malicious traffic

The malicious network traffic generated is divided into eight different scenarios, each representing a specific attack strategy to simulate realistic cyber threats. The attacker, who is located on the Kali-User and Kali-Remote devices within the user and remote LAN, executes the attacks in a modular, step-by-step manner. Each step builds on the previous one, reflecting the progressive nature of real-world cyberattacks. This structured methodology captures the sequential dependencies of multi-stage attacks such as reconnaissance, exploitation, and disruption, enabling detailed analysis of individual attack steps or the entire attack flow. A pause of approximately five minutes is built in between each step, allowing the attacker to analyze the results of the previous step before continuing.

### 4.4 Scenario 1

This scenario illustrates an attempt to disrupt business operations by slowing or denying access to a business-critical service. Here, the attacker targets the web server, which is a vital asset to the organization's operations. The steps in this scenario are shown in Table 4.

**Table 4:** Scenario 1 attack sequence.

SC1 sequence
DNS exploitation
5 min pause
NMAP mapping
5 min pause
DoS Hulk

The scenario starts with two attackers: one on the Remote User's LAN and one on the User's LAN. Both exploit the DNS configuration of the environment, which resolves the domain name of the web server as “web.sigen.net”. This allows the attackers to identify the corresponding IP address of the server. Next, the attackers use NMAP [10] to scan the target and find the port on which the web service is running. With this information they launch a Hulk [11] HTTP flood. The remote attacker initiates their steps as the Kali-User is in the middle of the attack, flooding the server with a massive amount of traffic. The overwhelming influx of requests causes the server to experience severe performance degradation, potentially rendering the service completely inaccessible.

### 4.5 Scenario 2

Similar to Scenario 1, this scenario illustrates an attempt to disrupt business operations by either slowing or blocking access to a critical service, in this case the web server, for a long period of time. The sequence of events in this scenario is shown in Table 5.

**Table 5:** Scenario 2 attack sequence.

SC2 sequence
DNS exploitation
5 min pause
NMAP mapping
5 min pause
DoS Slowloris

As in Scenario 1, two attackers are involved: one on the Remote User's LAN and one on the User's LAN. Both exploit the environment's DNS configuration, which resolves the web server's domain name to “web.sigen.net,” allowing the attackers to obtain the server's IP address. The attackers then use NMAP to scan the target and identify the port on which the web service is running. Once this information is obtained, they launch a Slowloris attack using Metasploit [12], which keeps connections open for long periods of time to exhaust server resources. The remote attacker initiates his part of the attack in the middle of the Kali-User actions, overwhelming the server with a large volume of traffic. This surge of traffic causes severe performance degradation, potentially rendering the service completely inaccessible.

### 4.6 Scenario 3

This scenario illustrates an attempt to disrupt business operations by slowing or blocking access to a critical service. The attacker focuses on the web server, which is a key asset for the organization to function. The sequence of actions in this scenario is shown in Table 6.

**Table 6:** Scenario 3 attack sequence.

SC3 sequence
DNS exploitation
5 min pause
NMAP mapping
5 min pause
DoS UDP

The attacker, located on the User's LAN, exploits the environment's DNS configuration, which resolves the Web server's domain name to “web.sigen.net”. This allows the attacker to determine the IP address of the server. The attacker then uses NMAP to scan the target and identify the port on which the web service is running. With this information, they initiate a UDP flood attack using Hping3 [13], which compromises the server with a high volume of traffic.

### 4.7 Scenario 4

This scenario depicts an attempt to disrupt the normal operations of an organization by degrading or blocking access to a critical service. As previous scenarios, the attacker targets the web server, which is an essential asset for the organization's day-to-day activities. The series of steps involved in the attack is shown in Table 7.

**Table 7:** Scenario 4 attack sequence.

SC4 sequence
DNS exploitation
5 min pause
NMAP mapping
5 min pause
DoS ICMP

The attacker, located on the User's LAN, exploits the DNS configuration that resolves the Web server's domain name to “web.sigen.net”, allowing the attacker to find the IP address of the server. With this information the attacker uses NMAP to scan the target and identify the port on which the web service is running. Once this is discovered, the attacker launches an ICMP flood attack using Hping3, flooding the server with a massive amount of traffic.

### 4.8 Scenario 5

This scenario illustrates an attempt to disrupt the operation of an organization by limiting or preventing access to a critical service, the web server. The sequence of actions in this attack is detailed in Table 8.

**Table 8:** Scenario 5 attack sequence.

SC5 sequence
DNS exploitation
5 min pause
NMAP mapping
5 min pause
DoS Push&Ack

Following a pattern similar to the previous scenarios, the attacker, positioned on the User's LAN, exploits the DNS configuration to identify the IP address of the web server. Using this information, the attacker performs an NMAP scan to locate the port on which the web service is running. Once the port is identified, the attacker initiates a Push&Ack flood attack using Hping3, flooding the server with an overwhelming amount of traffic.

### 4.9 Scenario 6

This scenario outlines an attack that uses a bruteforce SMB attack to test credentials in order to gain unauthorized access to network resources. By systematically trying a large number of potential login combinations, the attackers attempt to identify valid credentials that will allow them to escalate privileges, move laterally within the network, and ultimately compromise critical systems or sensitive data. The sequence of actions in this scenario is shown in Table 9.

**Table 9:** Scenario 6 attack sequence.

SC6 sequence
DNS exploitation
5 min pause
NMAP mapping
5 min pause
Bruteforce SMB

Similar to previous scenarios, the attacker begins by identifying the IP address of the DNS server. During an NMAP scan, they discover that the DNS server has an active SMB service running. Taking advantage of this, the attacker then deploys Hydra [14], a password cracking tool, using a dictionary of the 10,000 most common passwords [15]. By bruteforcing AD credentials, the attacker can gain unauthorized access to the machine, which opens the door for further malicious activity within the network.

### 4.10 Scenario 7

This scenario depicts an attack designed to gain unauthorized access to a computer and its files. Such access could expose sensitive data or serve as a first step to further exploitation, such as malware injection, ransomware, or insider information gathering. The detailed steps are shown in Table 10.

**Table 10:** Scenario 7 attack sequence.

SC7 sequence
DNS exploitation
5 min pause
NMAP mapping
5 min pause
Bruteforce SSH

Similar to the previous scenarios, the attacker first determines the IP address of the machine hosting the web site. During an NMAP scan, the attacker discovers an active SSH service on the target. Using this discovery, they deploy Hydra to try and crack the password, using a dictionary of the 10.000 most commonly used passwords. By bruteforcing the SSH credentials, the attacker can gain unauthorized access to the machine, setting the stage for further malicious activity.

### 4.11 Scenario 8

Similar to scenario 7, this scenario involves attacking a server to gain unauthorized access to sensitive files. Like so, the breach could lead to subsequent malicious actions such as injection of malware or deploying ransomware. In addition, the attacker could gain critical insider knowledge, such as database credentials or system configurations. The steps are shown in Table 11.

**Table 11:** Scenario 8 attack sequence.

SC8 sequence
DNS exploitation
5 min pause
NMAP reconnaissance + mapping
5 min pause
Bruteforce SSH + FTP

Following a pattern similar to the previous scenarios, the attacker first identifies the IP address of the Web server. Knowing that this server is part of the DMZ LAN, they use NMAP to scan the LAN for active machines and services. Among the devices identified, an FTP server that hosts both FTP and SSH services stands out. The attacker then performs bruteforce attacks on both services using a password dictionary containing the top 10.000 commonly used passwords and Hydra. These attacks could provide access to sensitive data or a leverage point for further compromises.

### 4.12 Feature extraction

Concerning the raw network packets generated from the previously described traffic generation steps, HERA [16], a tool designed to streamline the creation of flow-based datasets, was selected to aggregate these packets into flows and extract the features to construct the final dataset. An advantage this tool provides is the possibility of generating flows in different intervals, specifically the frequency in seconds at which a flow will be generated as long as there is still on-going activity in the flow during the specified time. This is a useful functionality when considering smaller intervals, such as the minimum of 1 s, will provide more updates on the flow while a higher number will generate fewer flows and allow for more summarization.

HERA was used to generate different versions of the dataset with flow intervals of 5, 10, 30 and 60 s, and then saved them in a CSV file format, where each line represents a network flow with multiple extracted features that summarize network statistics. Then, the same tool was used to label the dataset, categorizing each flow as either benign or belonging to a specific attack type as identified in the previous sections. Regarding the labeling process, it relied on a ground truth file that was carefully constructed to contain identifiable information about each attack instance that would allow us to properly classify the traffic. This included timestamps marking the beginning and end of the attack, IP addresses, port numbers, and the relevant protocols. To ensure the accuracy of the ground truth and to ensure that a reliable dataset was produced, a multi-step validation process was conducted. First, the attack scenarios were reviewed to confirm the expected malicious activity. Then, a thorough analysis of the raw traffic within the capture files was performed, cross-referencing known attack behavioral patterns to identify malicious traffic. Table 12 presents a summarized view of the traffic statistics found in the dataset.

**Table 12:** Dataset statistics.

Flow Volume Metrics		5 s	10 s	30 s	60 s
Total number of packets		37.681.001			
Total number of bytes		2.737.930.223			
Flows per label	dos-udp	786.432	393.216	131.072	65.536
	dos-icmp	786.432	393.216	131.072	65.536
	dos-pushack	785.472	392.736	130.942	65.806
	dos-slowloris	255.311	168.116	85.212	51.729
	dos-hulk	93.938	67.821	51.327	47.033
	recon-nmap	27.713	27.713	27.713	27.713
	benign-background	20.649	17.180	12.810	10.286
	benign-users	20.355	18.048	15.641	12.851
	bruteforce-ssh	12.388	8.677	4.696	4.688
	bruteforce-smb	10.002	10.001	10.001	10.001
	benign-admin	4.112	4.096	4.083	4.013
	bruteforce-ftp	3.344	3.344	3.344	3.344
	recon-dns	20	20	20	20
Total number of flows		2.806.168	1.504.184	607.933	368.556

## Limitations

Despite the careful design of this dataset, it is important to acknowledge its limitations. Since the performed cyberattacks were restricted to specific machines, expanding the scenarios to include more types of attacks across different network protocols and more LANs could improve data diversity. Additionally, even though the dataset includes benign network activity, it does not fully capture the variability observed in complex computer networks. Combining this dataset with real-world benign traffic generated by different user behaviours across multiple applications and device types could improve the generalization of machine learning and deep learning models.

## Ethics Statement

The authors confirm that they have read and followed the ethical requirements for publication in Data in Brief. This work does not involve human subjects, animal experiments, or any data collected from social media platforms.

## CRediT Author Statement

**Miguel Silva**: Methodology, Data curation, Validation, Investigation, Writing. **Daniela Pinto**: Methodology, Data curation, Validation, Investigation, Writing. **João Vitorino**: Conceptualization, Methodology, Validation, Investigation, Writing. **José Gonçalves**: Methodology, Validation, Investigation, Writing. **Eva Maia**: Conceptualization, Validation, Investigation, Supervision. **Isabel Praça**: Conceptualization, Resources, Project administration, Funding acquisition.

## Data Availability

ZenodoGeNIS: GECAD Network Intrusion Scenarios (Original data).
